# University College Hospital

**Published:** 1889-10-12

**Authors:** 


### UNIVERSITY COLLEGE HOSPITAL.

Mr. Godlee introduced a chapter of ancient history. During the seige of Turin, in 1539, a soldier was wounded in the arm, and being despaired of by the attending surgeon, came under the care of Ambrose Parè, who was then introducing the ligature, instead of boiling oil for the arrest of hæmorrhage. Even so lately as twenty years ago, limbs were amputated which are now saved. The importance of modern improvements in surgery can scarcely be over-rated, especially antisepticism and anæsthesia. The life of the student was next glanced at, who is a very different person to the medical student of former days, there being no doubt raised average general attainments before professional work is entered upon.

